# Spin-Crossover Nanoparticles in Electrospun Polymers: A Route to Bistable Materials for Smart Textiles

**DOI:** 10.1021/acsanm.5c02764

**Published:** 2025-08-06

**Authors:** Aleksandra Pacanowska, Alejandro Regueiro, Miguel Clemente-León, Eugenio Coronado, Alicia Forment-Aliaga, Magdalena Fitta

**Affiliations:** † 113066 Institute of Nuclear Physics Polish Academy of Sciences, Radzikowskiego 152, 31-342 Kraków, Poland; ‡ Instituto de Ciencia Molecular, Universitat de València, Catedrático José Beltrán 2, 46980 Paterna, Spain

**Keywords:** electrospinning, spin crossover, nanoparticles, composites, bistability

## Abstract

Developing strategies that transform crystalline molecular materials into processable forms is crucial for enabling their manipulation and integration into devices. This challenge is particularly relevant for bistable systems such as spin-crossover nanoparticles, which are often difficult to handle. Embedding these nanoparticles into organic polymers has emerged as a promising way to overcome these limitations. In this work, we investigated a series of iron­(II) triazole-based spin-crossover nanoparticles with the size of 37.0 ± 5 nm (**1)**, 55.4 ± 9.7 nm (**2**), and 116.8 ± 15.5 nm (**3**) incorporated into electrospun PVP fibers. Magnetic susceptibility measurements demonstrated that the hysteretic spin transition is preserved in all composites, with a significant broadening of their coercive fields. The most significant effect is observed in the cooling mode of the composites based on nanoparticles of bigger size, **2** and **3**, which is shifted to lower temperatures compared to their powder counterparts. A contrasting analysis of electrospun fibers and drop-casted films highlighted enhanced magnetic hysteresis and improved fiber stability, indicating a matrix geometry effect on spin crossover behavior. These findings underscore the potential of electrospun spin-crossover composite materials not only for creating flexible and scalable functional fabrics but also for precisely tailoring magnetic properties and enhancing robust spin-crossover behavior.

## Graphical abstract

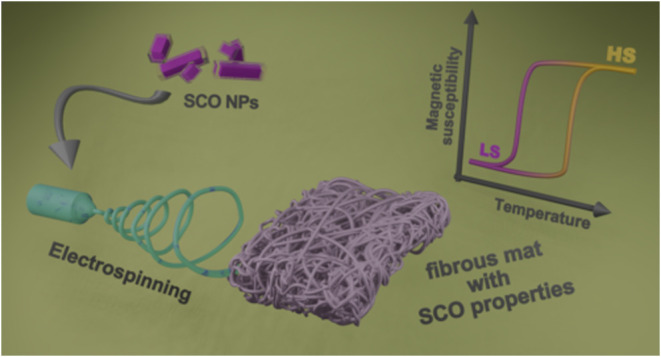

## 1 Introduction

Spin-crossover (SCO) phenomena constitute one of the most spectacular examples of molecular bistability.^1–5^ SCO complexes can be reversibly switched between two distinct states (high-spin and low-spin) by various external stimuli such as light, temperature, pressure, electric field, or the presence of certain analytes.^3^ This entropy-driven process is observed in d^4^–d^7^ metal complexes, although most of the examples are provided by Fe­(II) complexes, which undergo SCO transition between the diamagnetic low-spin state (LS, S = 0) and the paramagnetic high-spin state (HS, S = 2).^6–9^ This spin transition may be cooperative and is often accompanied by a thermal hysteresis at relatively high temperatures.^10,11^ The SCO transition also includes drastic changes in the volume of the molecular solid (changes greater than 10%),^12,13^ as well as in other electronic properties such as color^14^ and dielectric constant.^15^

All these properties offer great potential for various devices, such as sensors,^16,17^ actuators,^1,18–20^ and information units.^21^ To enhance the adaptability of molecular switches for devices, an advantageous approach involves the use of composite materials, particularly incorporating an organic polymer matrix.^2,22^ By integrating particles with precisely defined sizes, shapes, and spin-dependent characteristics into the polymer matrix, the resulting material often combines the properties of both the filler and the matrix. This approach allows the production of larger samples without increasing the amount of switching molecules, providing flexibility and enhanced stability over time. A notable success in this realm is the synergy observed in SCO/organic polymer hybrids, which demonstrate significant actuation amplitude and stress during the spin transition.^23^ This breakthrough holds promise for applications in biomimetic artificial muscles.^24^ Despite notable advancements, the realization of fully functional devices operating at the micro/nanoscale and room temperature remains a challenge. The effective use of these materials depends heavily on developing robust fabrication methodologies that enable the integration and handling of devices without compromising their bistability.

Among the extensively studied SCO materials, [Fe­(Htrz)_2_(trz)]_n_(BF_4_)_n_ (Htrz = 1,2,4–1H-triazole) stands out as a noteworthy example. It is a 1D coordination polymer exhibiting robust and broad hysteresis near room temperature. Notably, this material offers the advantage of being fabricated as nanoparticles (NPs) with finely tuned sizes while still retaining its memory effect.^25–27^ Additionally, they can be combined with polymers to enhance their processability. An intriguing example is the fabrication of actuators with thermally induced spin transition capabilities, using these spin crossover particles with an average size of 85 nm, dispersed within an SU-8 polymer matrix.^28^ This composite material was spray-coated onto silicon microcantilevers, demonstrating well-reproducible actuation upon thermal stimulation.

Among the various methods for composite preparation, electrospinning stands out as a particularly promising technique.^29^ The substantial elongation forces generated during fiber stretching circumvent issues commonly encountered in traditional preparation methods like solvent casting. A basic setup involves a syringe needle connected to a high-voltage power supply, a syringe pump, and a grounded collector. Electrospinning methods offer a versatile approach for creating polymer/molecule-based magnetic NP composites, generating fibers with diameters spanning from nano- to micrometers. This technique stands out for its simplicity, cost-effectiveness, and capacity to produce materials with substantial surface area-to-volume ratios, applicable across various material types. Electrospinning has been used to produce SCO-polymer composite fibers either from solutions or from particle–polymer suspensions. However, only a few successful examples have been reported thus far.^30,31^ It is worth noting that while alternative fiber fabrication methods, such as pressurized gyration, have also emerged, no SCO/organic polymer composites have been reported using this technique to date.^32–34^

The effective introduction of homologues of Fe-triazole coordination chain into the polymer matrix was confirmed by Mossbauer^30,31,35^ or Raman spectroscopies^36^ only at room temperature, without studying SCO transitions at varied temperatures. Interestingly, it was shown that soluble SCO material ([Fe­(NH_2_-trz)_3_]­(2 ns)_2_; NH_2_-trz = 4-amino-1,2,4-triazole; 2 ns = 2-naphthalenesulfonate) has been introduced in different polymer matrices.^35^ However, after the electrospinning process, only when using polyacrylonitrile most of the Fe­(II) centers of the compound remain in the LS state. In the case of other polymers (poly­(methyl methacrylate) or polyvinylpyrrolidone), Mossbauer spectra at room temperature showed a very low amount of original Fe­(II) in the LS state, being the SCO active centers.

In this context, the current study focuses on the synthesis of functional materials through the preparation of electrospun fibers composed of spin-crossover molecular materials and organic polymers. The primary goal is to create a system that maintains the filler’s switchable magnetic properties while preserving the organic polymer’s mechanical integrity. Additionally, the electrospinning technique offers precise control over the diameter and length of the fibers, enhancing the material’s versatility. Polyvinylpyrrolidone (PVP) was selected as the polymer matrix due to its solubility, ability to form fibers, and environmentally friendly nature. Its glass transition temperature, around 180 °C, is a key factor ensuring the composite material’s stability within operational temperature ranges. As the SCO filler, NPs of Fe­(Htrz)_2_(trz) coated with a SiO_2_ shell were employed. Notably, this nanostructuration offers adjustable sizing through synthetic modifications. Additionally, its easy redispersion capability, facilitated by sonification and magnetic stirring, and its robustness add to the system’s versatility and ease of use.

The overarching objective of this work is to explore the potential of such hybrid materials for use in smart fabrics: flexible, responsive textile systems capable of sensing and reacting to external stimuli. By integrating bistable SCO components into mechanically robust polymeric fibers, we aim to demonstrate a potential pathway for the development of future wearable technologies.

## 2 Experimental Section

### 2.1 Materials

All used chemical reagents, 1,2,4–1H-triazole (Sigma-Aldrich), iron tetrafluoroborate hexahydrate (Sigma-Aldrich), n-hexanol (Sigma-Aldrich), cyclohexane (Sigma-Aldrich), Triton X-100 (Sigma-Aldrich), tetraethyl orthosilicate 98% (Sigma-Aldrich), acetone (POCH), ethanol (POCH), polyvinylpyrrolidone *M*_w_ = 360 000 (PVP 360, Sigma-Aldrich), methanol (Chempur) were purchased and used without further purification.

### 2.2 Synthesis of Nanoparticles

NPs have been synthesized using the reverse micelle method: mixing the metal (Fe^II^) and the ligand (HTrz) in addition to tetraethyl orthosilicate (TEOS) (aqueous phases), with their respective organic phase – cyclohexane with Triton x-100 acting as surfactant and *n-*hexanol as cosurfactant. Once micelles are formed, both organic phases are mixed and left stirring, where the micelles will react to each other, bounded by the walls of the micelle, giving rise to NPs@SiO_2_. In turn, by changing the proportions of the precursors and the stirring time, NPs of different sizes have been prepared (details of synthesis in Table S1 in the Supporting Information).^27^

### 2.3 Pure PVP Fibers

PVP 360 powder (0.456 g) was dissolved in methanol (4 mL) and put on the gyromixer for 24 h in order to obtain a homogeneous solution of 12 wt % PVP/MeOH (Table S2). Then, the solution was introduced into the plastic syringe. The electrospinning was performed by use of SKE E-Fiber EF050 equipment. The inner diameter of a metal needle connected to the syringe was 0.4 mm, and before use, the end of the needle was cut to a length of approximately 1.1 cm and then sanded. The flow rate of the polymer solution was controlled by the syringe pump and equal to 1.5 mL/h. The solution of PVP without any fillers was electrospun with a voltage of 11 kV at room temperature with relative humidity ranging from 35–50% at a constant distance from the tip of the needle to the grounded collector of 10.5 cm. The fibers were collected as randomly oriented fiber mats on a metallic plate collector.

### 2.4 FeTrz/PVP Composite Fibers

Example – for **2**: In the first step of sample preparation, different amounts of NPs of **2** were weighted (15.5 mg; 48 mg; 110 mg; appropriately for **2–3.5%**; **2–10**% and **2–20%**) and homogeneously dispersed in methanol (4 mL) by sonification using an ultrasonication homogenizer probe (Sonoplus GM 4200, Bandelin electronic GmbH & Co. KG) operating at 30% power amplitude without pulsation for 2 min and magnetic stirring. Then, the PVP 360 powder (∼0.456 g) was introduced to the suspensions to give a solution of 12 wt % PVP/MeOH and immediately put on the gyromixer for 24 h to obtain a homogeneous suspension. The electrospinning was performed by use of SKE E-Fiber EF050 equipment. The composite suspension was introduced into the plastic syringe from which it was electrospun with the applied voltage of 13 kV at room temperature and relative humidity ranging from 35–50% and a constant flow rate of 1.5 mL/h. The fiber mats were randomly collected on a metallic plate collector with a distance between the tip and the collector equal to 10.5 cm. Scheme 1 illustrates the key steps involved in electrospinning fabrication, including: preparation of the nanoparticle–polymer suspension, the electrospinning process itself, and the formation of the final free-standing composite mat. A video demonstrating the process is provided in the Supporting Information.

**Scheme 1:**
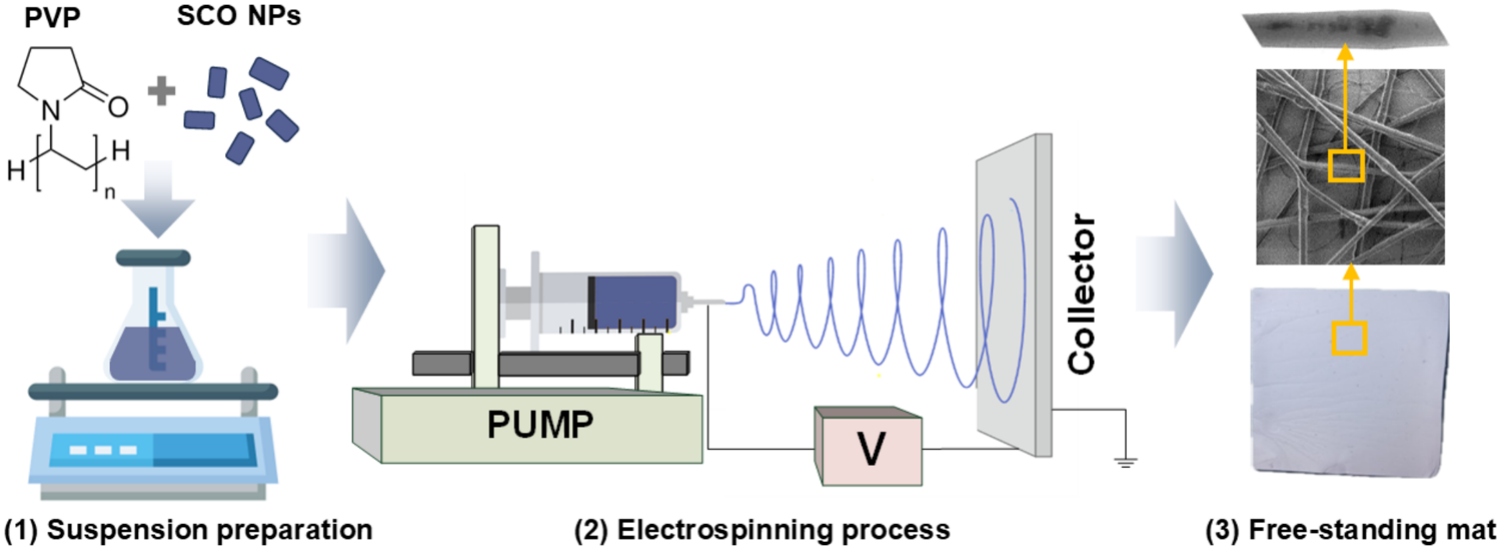
Schematic Illustration of the Complete Electrospinning Process, Depicting: (1) Suspension Preparation, (2) Electrospinning of the Prepared Suspension, and (3) Final Product of Electrospinning Process: Free-Standing Composite Mat

### 2.5 Characterization Techniques

#### 2.5.1 Magnetic Measurements

Magnetic data were collected with a Quantum Design MPMS XL-7. DC magnetic susceptibility measurements were performed under an applied magnetic field of 1 kOe in the temperature range between 300 and 400 K. Magnetic susceptibility measurements were performed using a heating/cooling rate of 1 K/min, except for the experiment investigating the effect of scan rate on the hysteresis width, which was carried out over a range of heating/cooling rates from 0.5 to 6 K/min to assess the dynamic response of the system. For the largest NPs (**3**) before the measurement, it was necessary to preheat the sample in the oven at 420 K for 30 min, in order to achieve a full transition from LS to HS. The diamagnetic contributions were corrected using Pascal tables. For the composite samples, the correction of magnetic susceptibility was performed using the Curie–Weiss law.

#### 2.5.2 Microstructure and Composition

Microstructure and composition analysis of fibers was performed using a Tescan Vega 3 scanning electron microscope equipped with an X-ray energy dispersive spectrometer EDAX Bruker. The composites were directly electrospun onto the Si (100) substrates in small amounts to minimize the charging effects during imaging. TEM imaging has been performed on the JEM-1010 operating at 100 kV. Samples of SCO NPs were prepared by dropping the suspensions of NPs on lacey Formvar/carbon copper grids (300 mesh). The SCO-filled composite fibers were directly electrospun onto the Cu grids with a Formvar/carbon layer. The size distribution of NPs and the diameters of composite electrospun fibers were determined with the use of ImageJ software. The nanoparticle size was determined by analyzing more than 70 individual particles from a series of TEM images. The average size and corresponding standard deviation, presented in the histogram in Figure S1 (Supporting Information), were calculated assuming a Gaussian distribution. Similarly, the fiber diameter distribution was evaluated based on measurements of over 400 fibers from multiple SEM images. The average diameter and standard deviation, calculated assuming a Gaussian distribution, are shown in the corresponding histogram (Figure S2).

#### 2.5.3 Calorimetry

Differential scanning calorimetry measurements were carried out at a scan rate of 10 K/min, in the heating and cooling modes using a Mettler Toledo DSC 821e model that operates in the temperature range −25 to 500 °C, equipped with a liquid nitrogen cryostat and a 200 W furnace. The temperature and enthalpy scales were calibrated by standard samples of indium and zinc. The measurements were carried out using about 5–18 mg of powdered and composite samples sealed in aluminum pans.

## 3 Results and Discussion

SiO_2_-coated SCO NPs of different sizes: 37.0 ± 5.0 nm (**1**), 55.4 ± 9.7 nm (**2**), and 116.8 ± 15.5 nm (**3**) (see ) were prepared following the previously reported method.^27^ In the initial phase of composite material preparation, the synthesized nanoparticles were redispersed in methanol as an organic solvent compatible with PVP. The silica shell provides stability and facilitates the dispersion in more polar solvents. In the next step, the polymer suspensions containing various initial filler concentrations (3.5, 10, and 20% by weight) were prepared. To maintain the high dispersity of the NPs and prevent their settling over time, suspensions were continuously agitated on a gyromixer. The freshly prepared polymer suspensions are characterized by a violet hue attributed to the color of the spin crossover fillers (see Figure S3).

A series of samples was fabricated using the electrospinning technique, with variations in NPs size and initial concentrations as determined during suspension preparation. While the composite mats predominantly exhibit a white appearance, notable differences arise in the sample with the highest initial concentration (20 wt %), where the electrospun mat visibly adopts a violet hue (Figure 1).

**Figure 1:**
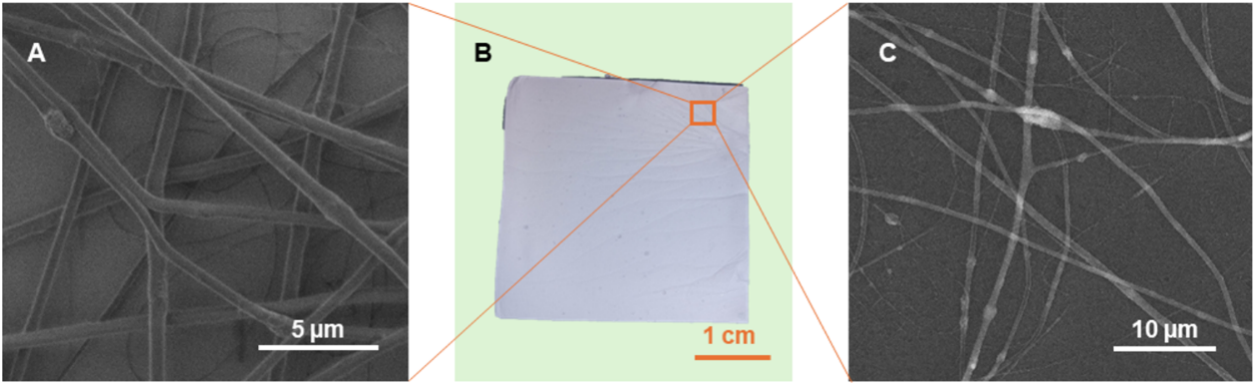
A photograph of the electrospun composite (B) and SEM images of the **2–20%** fibers recorded at 1 kV (A) and 20 kV (C).

Using scanning electron microscopy (SEM), it was determined that all mats consist of fibers with an average diameter of 793 ± 353 nm (see Figure 1). Upon closer examination, the fibers were found to consist of two distinct regions. The first region appears relatively smooth, with the surface of the single fiber remaining undisturbed. However, there are areas where the fiber diameter is slightly larger. Further observation under a transmission electron microscope (TEM, Figure 2) revealed that some of the NPs accumulate in these regions. Moreover, although not initially visible in SEM images, NPs are also present in the smooth regions, dispersing along the fibers without significantly changing their morphology. Therefore, TEM images reveal the localization of particles within the fibers, which is particularly prominent for the largest NPs with a rod-like topology. However, due to the significantly larger diameter of the fibers compared to the length of the NPs, their distribution within the fibers is nonuniform. This results in areas where NPs are situated in the middle of the fibers or shifted to the sides.

**Figure 2:**
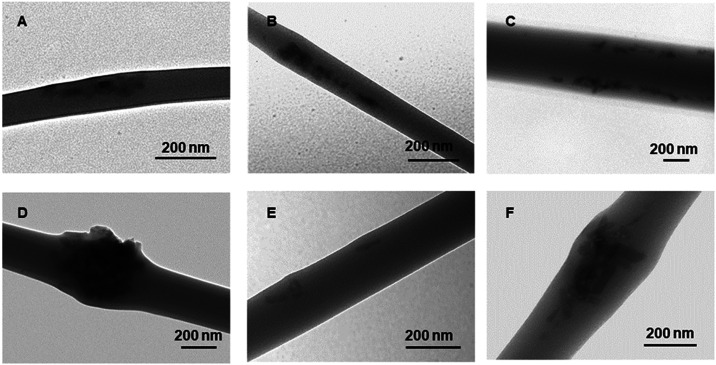
TEM images of electrospun composites: **1–10%** (A), **2–10%** (B, C), and **3–10%** (D, E, F).

As the size and initial concentration of the NPs increase, noticeable deformations in fiber production become evident. This phenomenon manifests by the formation of bubbles along fibers, which contain aggregated SCO filler, accompanied by disruption or breakage of the fiber surface. To attribute these bubble regions to the aggregation of SCO NPs, SEM imaging was conducted at a higher accelerating voltage (20 kV). Under these conditions, the Fe centers appear much brighter compared to the elements composing the organic polymer (C, N, and O). EDS mapping also confirmed that the bubble regions are much richer in the Fe metal centers compared to fiber strings (see Figures S4–S6).

Differential scanning calorimetry (DSC) was used to investigate the prevalence of SCO features in the fibers by tracking potential phase transitions associated with SCO (Figure 3). To ensure that features corresponding to PVP matrix in DSC analysis do not appear in the transition regions of the SCO compound, a control measurement was performed for pure PVP fibers (see Figure S7). Regarding pure NPs, **1** showed an exothermic HS→LS transition at 345 K upon cooling and an endothermic LS→HS transition at 376 K upon heating. DSC plots recorded for **2** revealed an exothermic peak at 349 K in the cooling and an endothermic peak at 375 K upon heating process, while for **3**, an exothermic peak was observed at 351 K and an endothermic peak at 382 K. Noteworthy, for all composite materials upon cooling and heating the DSC curves revealed similar peaks to those observed for pure NPs.

**Figure 3:**
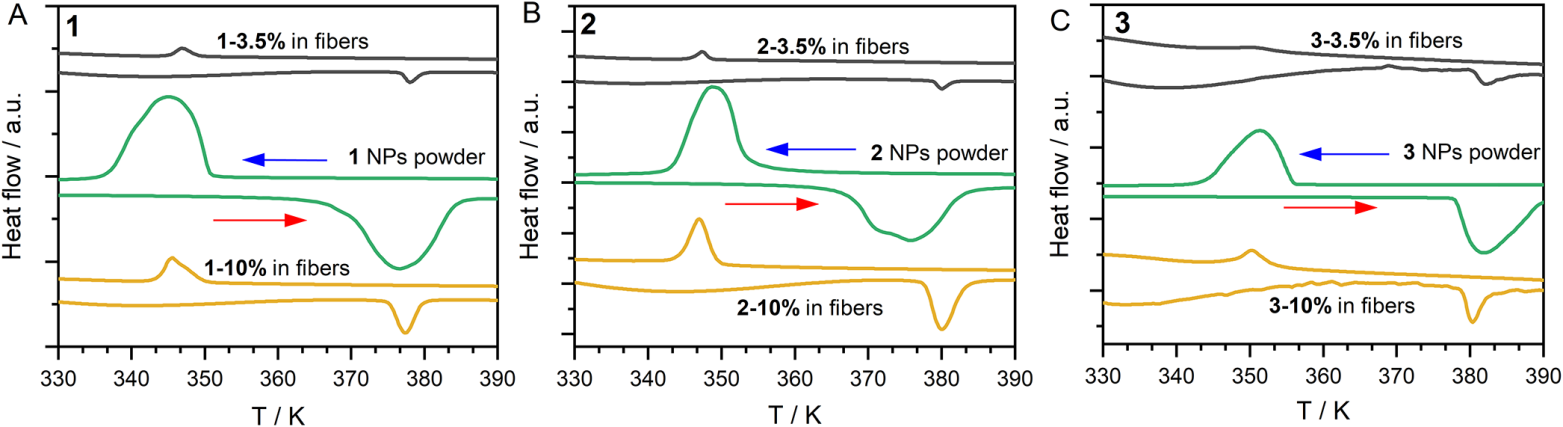
Comparison of DSC curves measured at a heating/cooling rate of 10 K·min^–1^ for (A) pristine **1** NPs and electrospun composites **1–3.5%** and **1–10%**, (B) pristine **2** NPs and electrospun composites **2–3.5%** and **2–10%**, and (C) pristine **3** NPs and electrospun composites **3–3.5%** and **3–10%**.

In parallel, the thermal dependence of the spin state of the SCO component in all composites was assessed by measuring their molar magnetic susceptibility (χ_m_) within the 300–400 K temperature range under a 1 kOe applied field. Multiple temperature cycles were measured to confirm repeatability. The initial two cycles exhibited slight differences attributed to solvent loss. However, after the third cycle, the magnetic data became consistent, indicating the attainment of a stable phase. For further discussion, we will utilize data from the fourth cycle for all examined samples. As shown in Figure 4, the NPs present a characteristic abrupt spin transition with a shift of a thermal hysteresis toward lower temperature upon reducing the NP size.

**Figure 4:**
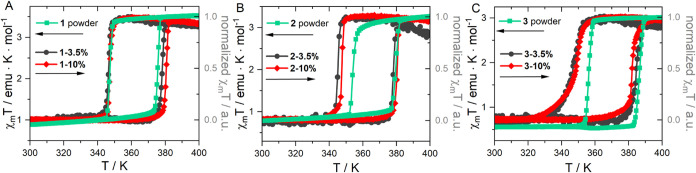
Thermal variation of χ_m_T measured with a heating/cooling rate of 1 K min^–1^ for **1**, **1–3.5%**, **1–10%** (A), **2**, **2–3.5%**, **2–10%** (B), and **3, 3–3.5%**, **3–10%** (C). For the composite samples, the correction of magnetic susceptibility was performed using the Curie–Weiss law.

Thus, as it is summarized in Table 1, the spin transition temperatures of the NPs in the heating mode *T*_1/2_(↑) are 375.6, 379.7, and 386.4 K while in the cooling mode *T*_1/2_(↓), the transition temperatures are 347.4, 354.1, and 356.3 K for samples **1**, **2**, and **3**, respectively. The χ_m_T product above 390 K for NPs reaches values of about 3.53 emu K mol^–1^(**1**), 3.27 emu K mol^–1^(**2**), and 3.01 emu K mol^–1^(**3**). For **1–10%** composite, *T*_1/2_(↑) = 380.6 K and *T*_1/2_(↓) = 346.5 K are observed, resulting in a ≈34 K thermal hysteresis (≈28 K for **1**). The broadening of the thermal hysteresis loops is observed in the **2**- and **3**-based composites too, with widths of approximately 33 K for both **2**-10% and **3**-10%. In comparison, the pure NPs **2** and **3** exhibit narrower hysteresis widths of about 25 and 30 K, respectively. The most significant effect is observed in the cooling mode of the composites based on NPs of bigger size, **2–3.5**, **2–10**, **3–3.5**, and **3–10**%, which is shifted to lower temperatures compared to their powder counterparts. Furthermore, the spin transition of the composite samples obtained from **3** becomes more gradual. Such a remarkable influence of the matrix on the spin-crossover properties of nanoparticles is rare. However, it has been previously observed in NPs of [Fe^II^(Htrz)_2_(trz)]­(BF_4_)^28^ and other SCO compounds^37–39^ in various matrices. This effect is often attributed to elastic interactions with the matrix, but more detailed studies are still needed.

**Table 1:** Summary of the Magnetic Properties of All NPs and Electrospun Samples Prepared in This Work and Other Published Data

sample	*T*_1/2_ (↑) (K)	*T*_1/2_ (↓) (K)	Δ*T* (K)	ref
**1** powder	375.6	347.4	28.2	this work
**1–10%**	380.6	346.5	34.1	
**1**–**3.5%**	378.2	347.0	31.2	
**2** powder	379.7	354.1	25.6	this work
**2**–**10%**	380.5	347.4	33.1	
**2**–**3.5%**	378.2	345.0	33.2	
**3** powder	386.4	356.3	30.1	this work
**3–10%**	382.1	348.5	33.6	
**3–3.5%**	384.6	348.1	36.5	
[Fe(NH_2_-trz)_3_]Cl_2_ powder	350	342	8	^31^
[Fe(NH_2_-trz)_3_]Cl_2_ in PMMA^a^	353	345	8	
[Fe(NH_2_-trz)_3_](2 ns^b^)_2_ powder	317	293	24	^31^
[Fe(NH_2_-trz)_3_](2 ns)_2_ in PMMA	315	296	19	
[Fe(4-octadecyl-1,2,4-triazole)_3_(ClO_4_)_2_]_n_ in aPS^c^	gradual transition		no hysteresis	^40^

a poly­(methyl methacrylate).

b 2-naphthalenesulfonate.

c atactic polystyrene.

The most important effect is illustrated in Figure 5, which shows the χ_m_T vs T plot for NPs **2** and **2-**based composites recorded in different temperature sweep rates to trace the change of transition temperature and hysteresis width. Typically, in SCO materials, wider hysteresis is observed at a faster scan rate.^41,42^ The effect of scan rate on the hysteresis width was investigated over the range of 0.5–6 K/min. For the NPs **2** powder samples, only slight changes in the shape of the χ_m_T vs T plots upon change of temperature sweep rate are observed. In contrast, the composite materials display thermal hysteresis loops widening as the scanning speed decreases, and a greater dependence on scan rate is observed in the heating branch (Table S3).

**Figure 5:**
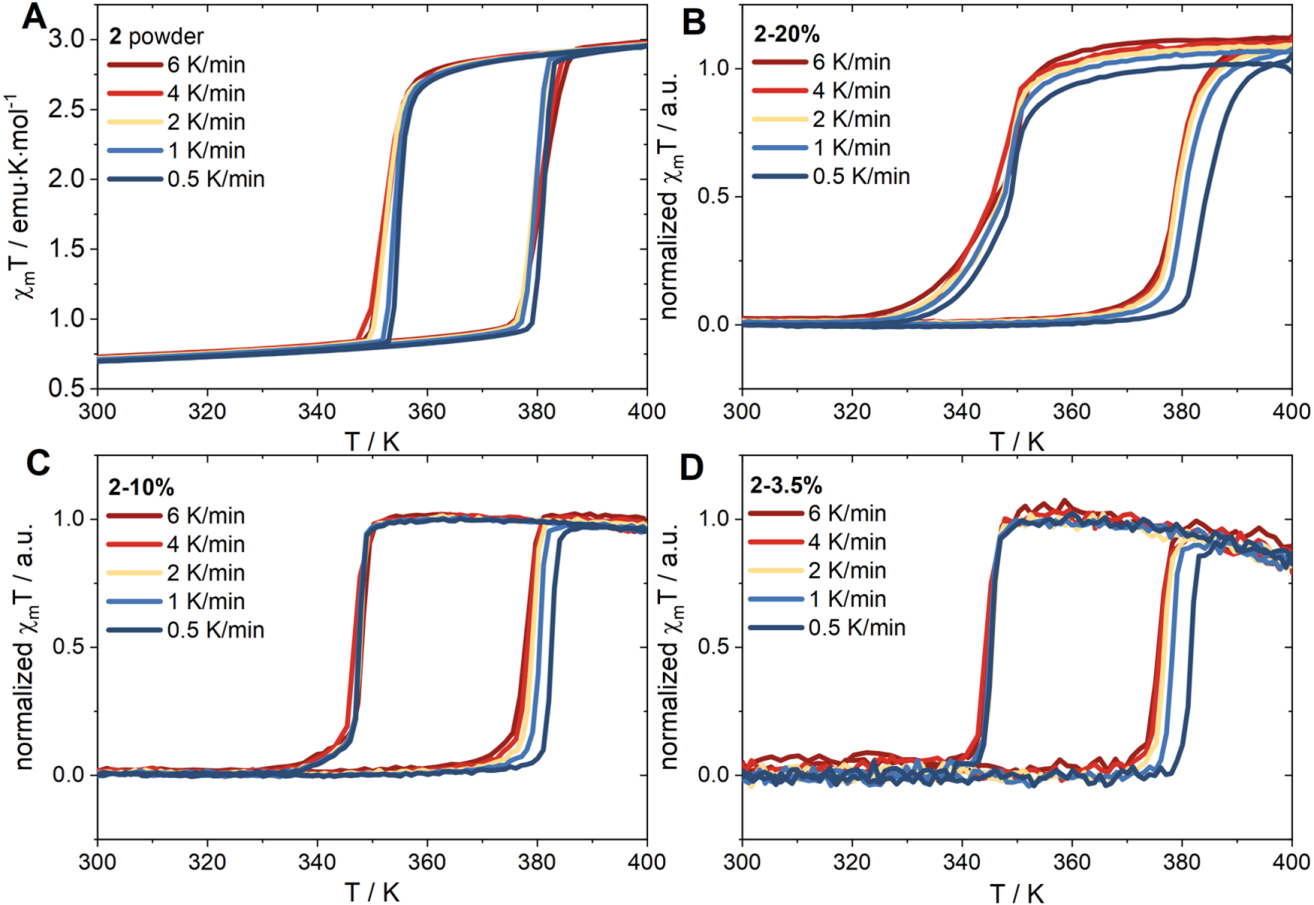
Temperature dependence of χ_m_T vs T at scan rates ranging from 0.5 to 6 K min^– 1^ measured for powder **2** (A), **2–20%** (B), **2–10%** (C), and **2–3.5%** (D).

To evaluate the stability of the SCO behavior, the aging effect was examined (Figure S9). Interestingly, encapsulating nanoparticles within electrospun fibers resulted in a more robust and stable spin-crossover response, in contrast to the suspensions used during the electrospinning process, which degraded over time (Figure S2).

As we have shown above, the incorporation of SCO NPs into electrospun PVP fibers maintains high cooperativity. To better understand the matrix effect, composite films were formed from the solutions used for electrospinning by drop-casting. Figure 6 shows the comparison of the magnetic susceptibility profile vs temperature measured for electrospun fibers and films obtained from the same solutions. The spin transition temperatures of composite films are shifted to a lower temperature, and the shape of thermal hysteresis shows a decrease in abruptness and, therefore, less effective spin transition. These results demonstrate that electrospun composite material has an enhanced magnetic hysteresis behavior compared to the drop-casted films.

**Figure 6:**
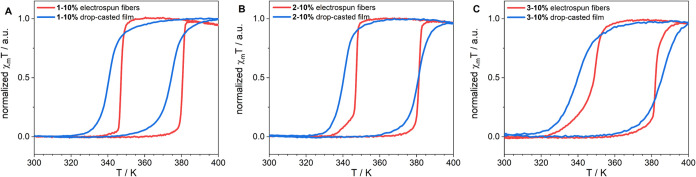
Χ_m_T vs T plots measured with a heating/cooling rate of 1 K min^–1^ for electrospun fibers and films obtained from the same solutions: **1–10%** (A), **2–10%** (B), and **3–10%** (C).

To the best of our knowledge, the magnetic properties of electrospun fibers with the SCO components have been investigated in two prior studies.^31,40^ In these works, composites were fabricated from solutions containing dispersed or dissolved SCO compounds and organic polymers. The studies of the fibers composed of a mixture of atactic polystyrene (aPS) and [Fe­(4-octadecyl-1,2,4-triazole)_3_(ClO_4_)_2_], revealed that the spin-transition temperature of the blend matches that of the pure [Fe­(4-octadecyl-1,2,4-triazole)_3_(ClO_4_)_2_]_n_.^40^ However, the transition in the blend is more gradual, and unlike the pure SCO material, the blend does not exhibit hysteresis in the spin transition. On the other hand, the investigation of the magnetic properties of a composite containing iron­(II) triazole complexes within a PMMA nanofiber matrix showed that the composite retained the SCO properties with minimal changes in hysteresis compared to the pure SCO complex.^31^ This slight shift indicates that the electrospinning process did not significantly alter the hysteresis. The only report describing composites containing presynthesized nanoparticles was based on the use of [Fe­(NH_2_-trz)_3_]­(BF_4_)_2_ and [Fe­(Htrz)_2_(trz)]­(BF_4_)­NPs in polylactic acid (PLA), and the spin transition was evaluated exclusively by Mössbauer studies. In this study, the spin crossover behavior of [Fe­(NH_2_-trz)_3_]­(BF_4_)_2_ compounds and their nanoparticles remains evident within the polymer matrix. At 77 K, only the LS state is observed, while a mixture of HS and LS states appears at 293 K. For both [Fe­(NH_2_-trz)_3_]­(BF_4_)_2_ and [Fe­(Htrz)_2_(trz)]­(BF_4_), the HS/LS ratio differs in polymer fibers compared to the bulk material. Notably, in both cases, the LS population increases in the composite material.^30^ This suggests that electrospinning influences the SCO fillers’ behavior, leading to a mixed-state presence in the LS temperature region, as supported by high-quality Mössbauer data.

Our results of the composites based on presynthesized [Fe­(Htrz)_2_(trz)]­(BF_4_) nanoparticles embedded in PVP electrospun fibers reveal similarities to other studies where spin crossover nanoparticles have been integrated into different polymer matrices. Examples include their incorporation in epoxy-based photoresist,^28^ micrometer-thick films in piezoelectric polymers,^43^ or silica-based xerogels.^38^ Interestingly, key features such as the widening of thermal hysteresis during magnetic measurements and the retention of a high degree of cooperativity are observed.

The choice of polymer matrix and solvent can influence the degree of SCO nanoparticle aggregation and clustering, particularly when the polarity of the components varies.^44^ The large differences in magnetic behavior observed between electrospun fibers and drop-cast films may result from interactions between the silica coating on the nanoparticles and the PVP polymer matrix. In previous studies on TEOS/PVP fibers, ATR-FTIR analysis highlighted structural differences between electrospun fibers and dried films. The electrospun fibers exhibited stronger bonding interactions and more organized silica networks, whereas the dried films retained more moisture and incomplete networks.^45^ These distinctions, largely driven by the efficiency of solvent evaporation during sample preparation, could potentially influence the SCO behavior of the embedded nanoparticles.

## 4 Conclusions

In summary, we demonstrated a straightforward method for fabricating SCO composite fibers by incorporating Fe­(II) triazole-based nanoparticles of defined sizes (37.0 ± 5 nm, 55.4 ± 9.7 nm, and 116.8 ± 15.5 nm) into electrospun PVP matrices. The resulting fibrous mats exhibited clear differences in magnetic behavior. SEM analysis confirmed nonuniform NPs distribution along the fibers, which influenced local morphology and, potentially, spin-transition cooperativity. Magnetic susceptibility measurements revealed well-defined, hysteretic spin transitions preserved in all composite samples, with an increase in coercive field width across all NPs sizes. Notably, the larger nanoparticles (∼55 and ∼117 nm) showed cooling-branch shifts of the transition to lower temperatures compared to their powder counterparts. The fibers also exhibited long-term magnetic stability.

A comparative analysis with drop-casted films prepared under similar NPs concentrations, highlighted significantly improved abruptness and thus a more effective spin transition in electrospun samples, suggesting that the fibrous matrix geometry and polymer-NP interaction play a critical role in modulating SCO behavior. This difference may stem from solvent evaporation kinetics and polymer distribution during film formation.

Overall, our results underscore the relevance of electrospinning as a scalable and tunable strategy for producing mechanically flexible, bistable hybrid materials. The ability to control spin-transition temperature, hysteresis width, and stability through NP size and composite architecture opens new avenues for integrating SCO materials into smart textile applications and responsive electronic platforms.

## Supplementary Material





## Data Availability

The data supporting this article have been included as part of the Supporting Information. Additional data related to this paper may be requested from the corresponding authors.

## Notes

TEM images of SCO NPs, experimental conditions for the preparations of nanoparticles and polymer solutions, photographs of electrospinning polymer suspensions, SEM images of composite mats and EDS map of C, Fe, and Si, DSC for pure PVP fibers and composites, comparison of the χ_m_T­(T) plots for electrospun fibers: for freshly prepared samples and after 4 months, summary of the magnetic properties of NPs and their electrospun counterparts (PDF)
Video demonstrating the process of suspension preparation and electrospinning (AVI)
