# Supplementary material for: Spin-Crossover Nanoparticles in Electrospun Polymers: A Route to Bistable Materials for Smart Textiles
Source: ACS Appl Nano Mater. 2025 Aug 6;8(32):15999–6007. doi: 10.1021/acsanm.5c02764 (PMC12362628; doi:10.1021/acsanm.5c02764)
Supplement: Supplementary file 1 [file an5c02764_si_001.pdf]

Supporting Information

Spin-Crossover Nanoparticles in Electrospun Polymers: A Route to Bistable Materials for Smart Textiles

Aleksandra Pacanowska[1], Alejandro Regueiro[2], Miguel Clemente-León[2], Eugenio Coronado[2], Alicia Forment-Aliaga[2*], Magdalena Fitta[1*]

[1]Institute of Nuclear Physics Polish Academy of Sciences, Radzikowskiego 152, 31-342 Kraków, Poland
[2]Instituto de Ciencia Molecular, Universitat de València, Catedrático José Beltrán 2, 46980 Paterna, Spain

Corresponding Authors:

E-mail: magdalena.fitta@ifj.edu.pl (Magdalena Fitta)
E-mail: alicia.forment@uv.es (Alicia Forment-Aliaga)

Table of Contents

Figure S1. TEM images of initial SCO nanoparticles: **1** (A), **2** (B), and **3** (C). The lower panel showcases the respective histograms showing size distribution of nanoparticles: **1** (D), **2** (E), and **3** (F), calculated with ImageJ software in a manner shown schematically in TEM images. The average NPs size and its standard deviation shown above histograms, have been calculated from Gaussian distribution.
Figure S2. SEM image of **2-10%** composite fibers (A) together with calculated size distribution of produced fibers' width (B), obtained with ImageJ software in a manner shown schematically in SEM image. For increased statistic the average width of fibers and the standard deviation shown above histogram, have been counted on several SEM images and calculated from Gaussian distribution.
Table S1. Experimental conditions for the preparations of nanoparticles.
Table S2. Experimental conditions for the preparations of polymer solutions.
Figure S3. Photographs of electrospinning polymer suspensions of **1**, **2,** and **3**.
Figure S4. SEM image of **1-10%** composite fibers and the corresponding EDS mapping of C, Fe, and Si.
Figure S5. SEM image of **2-10%** composite fibers and the corresponding EDS mapping of C, Fe, and Si.
Figure S6. SEM image of **3-10%** composite fibers and the corresponding EDS mapping of C, Fe, and Si.
Figure S7. DSC for pure PVP fibers.
Figure S8. DSC curves measured for **1-10%** (A, B) and **2-10%** (C, D) after 5 measurement cycles in the temperature range 300 – 400 K.
Figure S9. Comparison of the χ_mT vs. T plots for electrospun fibers: **1-10%** (A), **2-10%** (B), and **3-10%** (C) for freshly prepared samples (red curves) and after 4 months (brown curves).
Table S3. Summary of the magnetic properties of **2** NPs and their electrospun samples (**2-20%**, **2-10%** and **2-3.5%**) derived from temperature dependence of χ_mT vs T at scan rates ranging from 0.5 K min^{-1} to 6 K min^{-1}.

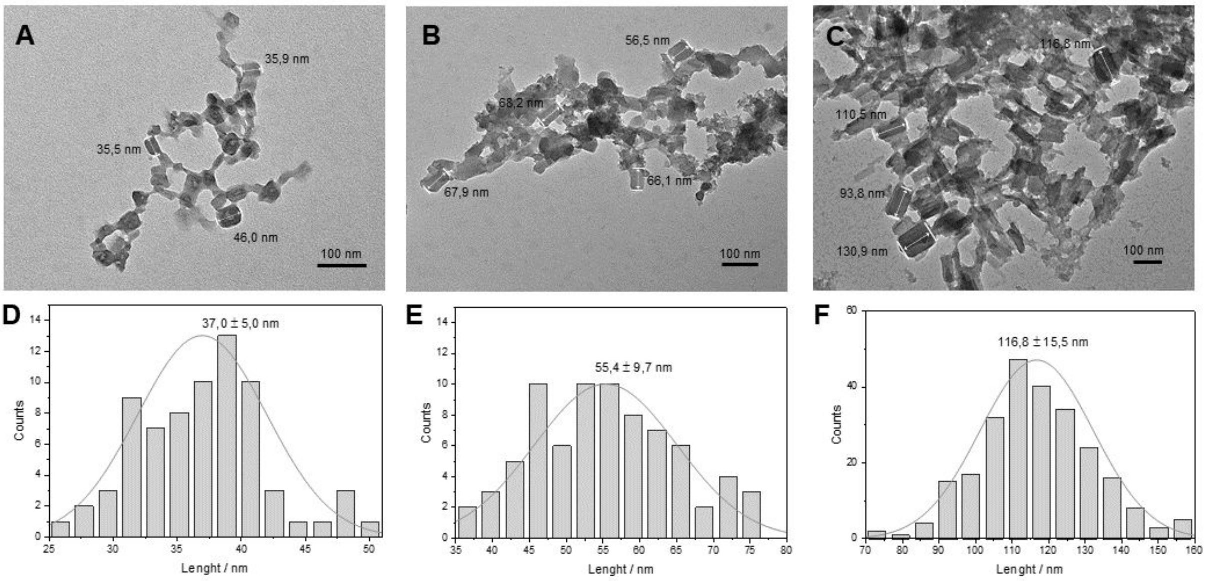

Figure S1. TEM images of initial SCO nanoparticles: **1** (A), **2** (B), and **3** (C). The lower panel showcases the respective histograms showing size distribution of nanoparticles: **1** (D), **2** (E), and **3** (F), calculated with ImageJ software in a manner shown schematically in TEM images. The average NPs size and their standard deviation shown above histograms, have been calculated from Gaussian distribution.

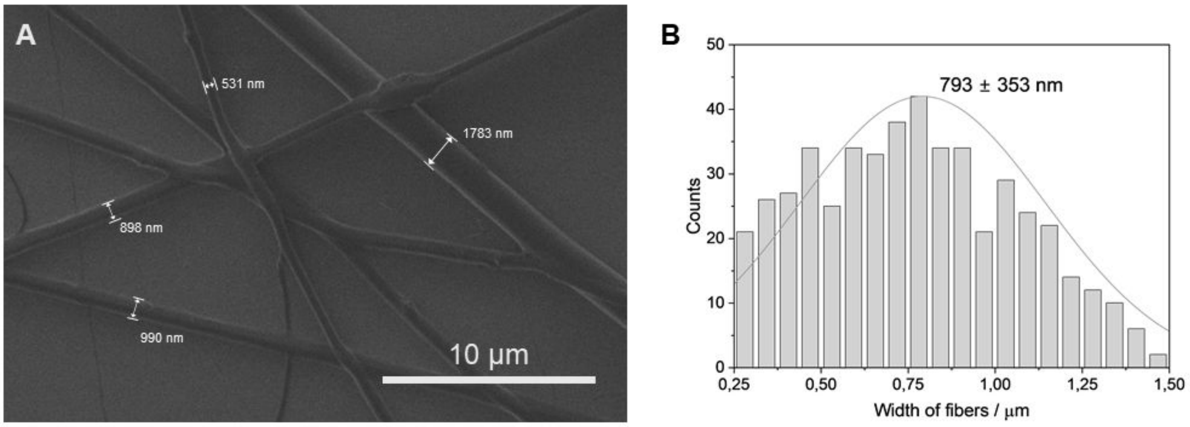

Figure S2. SEM image of **2-10%** composite fibers (A) along with calculated size distribution of fiber widths (B), obtained with ImageJ software as illustrated schematically in the SEM image. To improve statistical reliability, the average width of fibers and the standard deviation shown above the histogram were calculated on several SEM images and fitted to a Gaussian distribution.

Table S1. Experimental conditions for the preparation of nanoparticles.

Nanoparticles preparation	1	2	3
	Aqueous phase / each in 0.5 mL H_2O		
$Fe(BF_4)_2 \cdot 6H_2O$	253 mg	210 mg	180 mg
1,2,4-1*H*-triazole ligand	156 mg	130 mg	110 mg
tetraethyl orthosilicate (TEOS)	0.1 mL	0.1 mL	0.1 mL
	Organic phase		
Cyclohexane	7.5 mL	7.5 mL	7.5 mL
n-hexanol	2.7 mL	1.8 mL	1.8 mL
Trition X-100	2.7 mL	1.8 mL	1.8 mL
Synthesis time	2 h	12 h	48 h
Average length	37.0 ± 5.0 nm	55.4 ± 9.7 nm	116.8 ± 15.5 nm

Table S2. Experimental conditions for the preparation of polymer solutions.

Polymer suspensions	1		2			3	
SCO NP concentration	3.5%	10%	3.5%	10%	20%	3.5%	10%
Amount of SCO NP / mg	16.3	47.51	14.47	48.31	111.0	15.97	48.31
PVP 360 / g	0.459	0.455	0.454	0.454	0.455	0.455	0.459
Methanol / mL	4	4	4	4	4	4	4

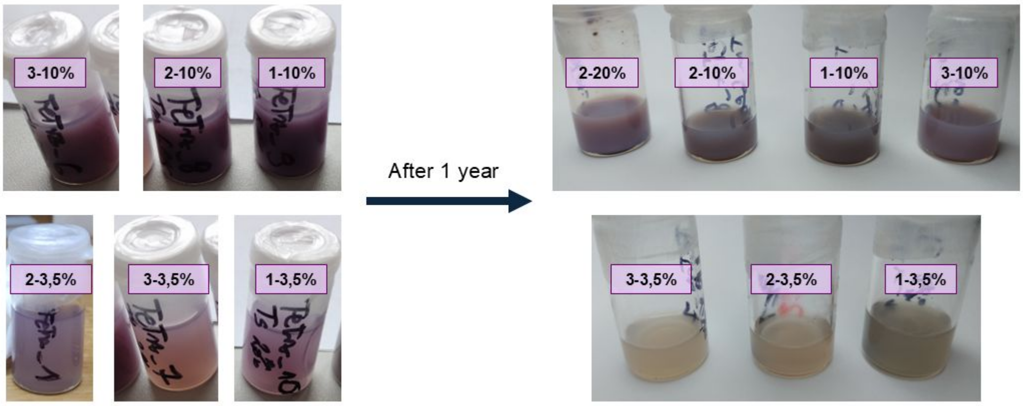

Figure S3. Photographs of electrospinning polymer suspensions of **1**, **2,** and **3** freshly prepared (left) and after a year (right).

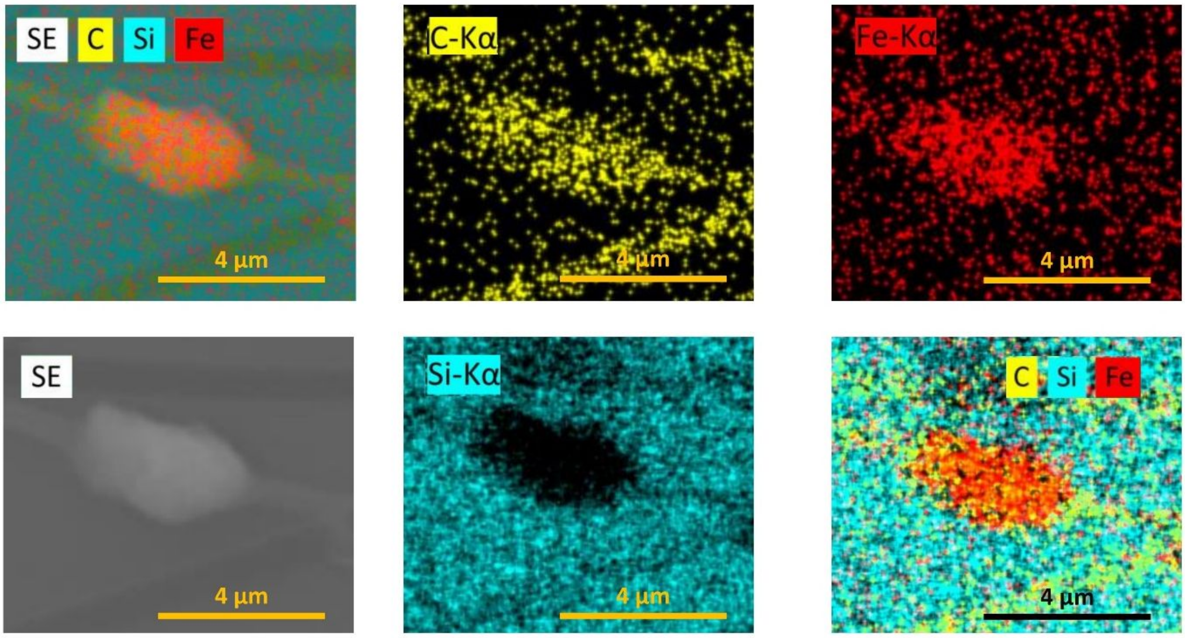

Figure S4. SEM image of **1-10%** composite fibers and the corresponding EDS mapping of C, Fe, and Si.

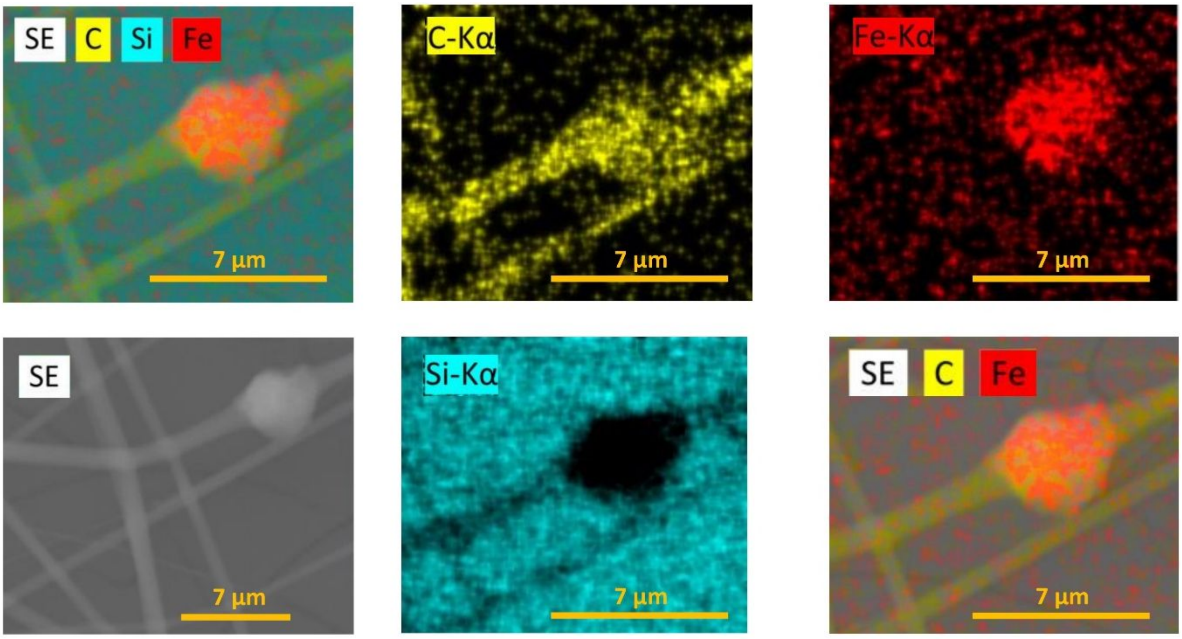

Figure S5. SEM image of **2-10%** composite fibers and the corresponding EDS mapping of C, Fe, and Si.

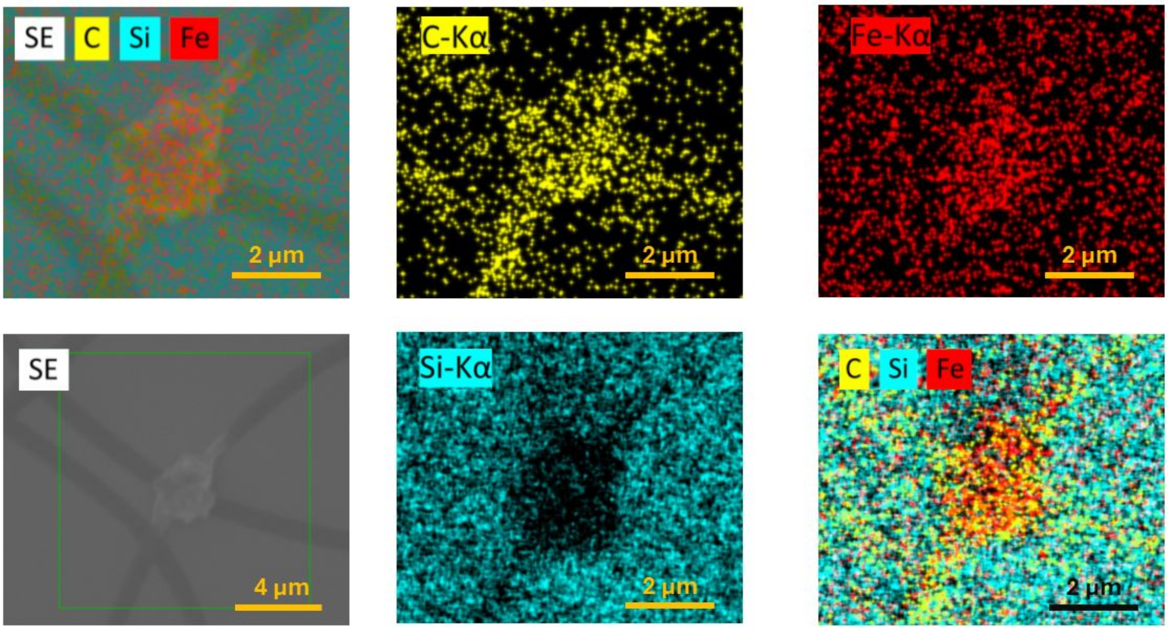

Figure S6. SEM image of **3-10%** composite fibers and the corresponding EDS mapping of C, Fe, and Si.

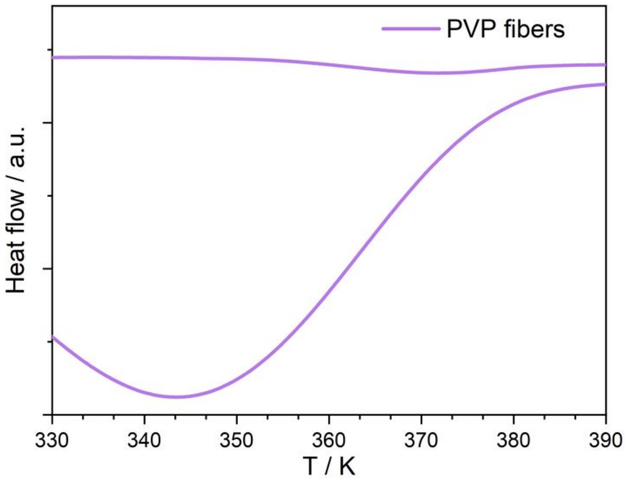

Figure S7. DSC curves in heating (lower curve) and cooling (upper curve) for pure PVP fibers. In the lower curve a noticeable widening is visible caused by the evaporation process of excess solvent from the freshly prepared electrospun fibers.

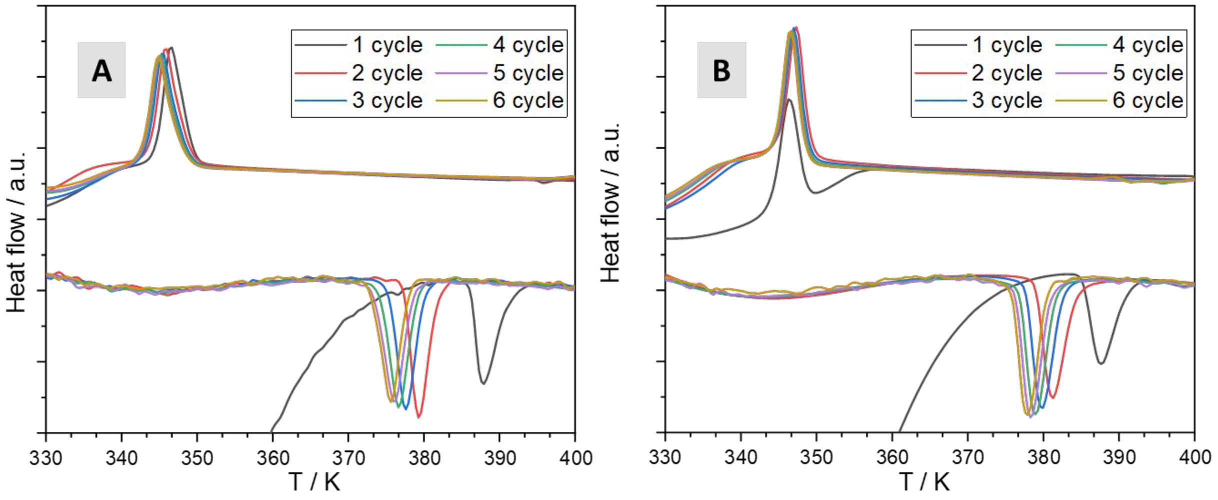

Figure S8. DSC curves measured for **1-10%** (A) and **2-10%** (B) after 6 measurement cycles performed in the temperature range 300 – 400 K to confirm the repeatability of SCO behavior in fibers. In both cases for the first cycle, the endothermic peaks upon warming exhibited a shift attributed to solvent loss. However, from the third up to the sixth cycle, the calorimetric data became consistent.

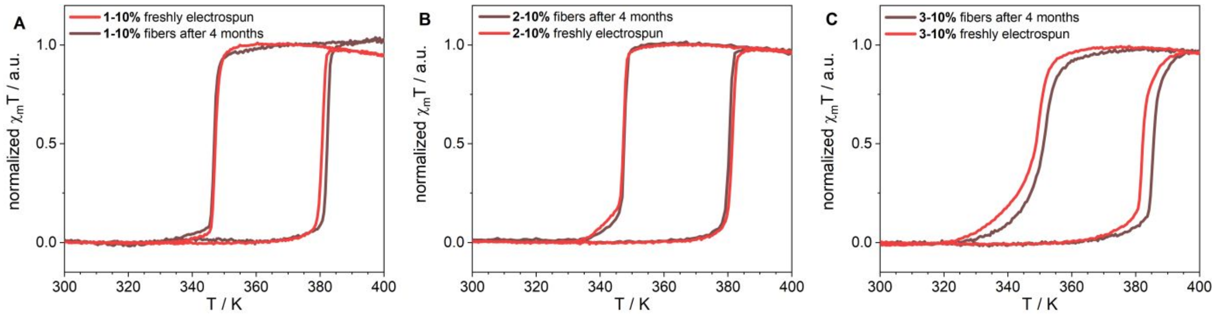

Figure S9. Comparison of the $\chi_m T$ vs. T plots for electrospun fibers: **1-10%** (A), **2-10%** (B), and **3-10%** (C) for freshly prepared samples (red curves) and after 4 months (brown curves).

Table S3. Summary of the magnetic properties of **2** NPs and their electrospun samples (**2-20%**, **2-10%** and **2-3.5%**) derived from temperature dependence of $\chi_m T$ vs T at scan rates ranging from 0.5 K min^{-1} to 6 K min^{-1}.

Sample	**Scan rate (K/min)**	**$T_{1/2}$ (↑) (K)**	**$T_{1/2}$(↓) (K)**	**Δ*T* (K)**
**2** powder	0.5	381.2	355.0	26.2
	1	379.7	354.1	25.6
	2	379.5	352.7	26.8
	4	380.5	352.5	28.0
	6	380.8	352.8	28.0
**2-20%**	0.5	384.7	348.6	36.1
	1	381.0	347.0	34.0
	2	379.5	346.0	33.5
	4	379.3	345.9	33.4
	6	379.3	346.1	33.2
**2-10%**	0.5	382.7	347.5	35.2
	1	380.5	347.4	32.9
	2	379.1	347.7	31.4
	4	378.5	346.5	32.0
	6	377.9	347.9	30.0
**2-3.5%**	0.5	381.5	345.3	36.2
	1	378.2	345.0	33.2
	2	376.7	345.3	31.4
	4	375.8	344.3	31.5
	6	375.8	345.0	30.8
